# The Precision Psychiatry Roadmap: towards a biology-informed framework for mental disorders

**DOI:** 10.1192/j.eurpsy.2025.68

**Published:** 2025-08-26

**Authors:** M. Kas

**Affiliations:** Groningen Institute for Evolutionary Life Sciences, University of Groningen, Groningen, Netherlands

## Abstract

**Abstract:**

Measuring biology is considered the cornerstone of diagnosis in medicine. For example, we have blood tests to diagnose auto-immune diseases, such as rheumatoid arthritis or lupus. Also, cancer patients with, for example, colon cancer, will receive personalized treatments following biomarker diagnostics of their tumor. In contrast, current classification methodologies for mental disorders are based on signs and symptoms. These classification systems provide a uniform language to assess psychiatric and neurological disorders across the globe. Clinicians use these diagnoses to communicate with their patients and with other clinicians, and to request, when applicable, reimbursement from payers. Unfortunately, currently available psychotherapies and pharmacotherapies work only in a small proportion of patients within these diagnostic categories. This clinical heterogeneity in treatment response within diagnostic categories is likely explained by biological variation between patients that is not being accounted for in the current classification system for mental disorders. In addition, there is transdiagnostic overlap allowing for novel opportunities to develop innovative quantitative biological endpoints and treatments beyond the current diagnostic framework. The European College of Neuropsychopharmacology (ECNP) New Frontiers meeting 2024 addressed these issues, and indicated a high need for a biology-informed framework to establish more precise diagnosis and treatment for mental disorders. The ECNP, following the 2024 New Frontiers Meeting, is coordinating a global initiative to design and implement a Precision Psychiatry Roadmap. By mobilizing resources and harmonizing translational methodologies and datasets, the aim is to discuss, design, and implement an iterative framework that incorporates biology-informed evidence into symptom-based syndromes, allowing for more discovery and implementation of mechanism-based effective treatments for mental disorders.

**Disclosure of Interest:**

None Declared

